# Healthcare Professionals’ Beliefs and Views towards Exercise during Pregnancy: A Cross-Sectional Study in Greece

**DOI:** 10.3390/healthcare12111089

**Published:** 2024-05-25

**Authors:** Vasileios Daglas, Nikolaos Kostopoulos, Irina Mrvoljak-Theodoropoulou, Aikaterini Lykeridou, Evangelia Antoniou

**Affiliations:** 1 Department of Midwifery, School of Health & Care Sciences, University of West Attica, 12243 Athens, Greece; klyker@uniwa.gr (A.L.); lilanton@uniwa.gr (E.A.); 2 School of Physical Education and Sport Science, National and Kapodistrian University of Athens, 17237 Athens, Greece; nikkosto@phed.uoa.gr; 3 Department of Psychology, National and Kapodistrian University of Greece, 15784 Athens, Greece

**Keywords:** exercise, pregnancy, healthcare professionals, beliefs, views, benefits, midwives, obstetricians, pregnant woman

## Abstract

Background: Healthcare professionals appear to play a key role in shaping pregnant women’s views and attitudes towards lifestyle issues, such as exercise. The aim of this study is to investigate the views and beliefs of Greek midwives and obstetricians regarding exercise during pregnancy. Methods: This is a cross-sectional study conducted during the period of January 2022–March 2023. For this study, 237 Greek midwives and obstetricians employed in healthcare settings in Attica, Greece completed an anonymous and self-report questionnaire. Four different/independent models of multivariate analyses of variance were conducted. Results: The vast majority of these healthcare professionals (88.6%) believed that exercise during pregnancy is generally beneficial. According to the multivariate analyses, healthcare professionals with postgraduate/doctoral studies were more likely to believe that (a) exercise is generally beneficial (*p* = 0.03), (b) pregnant women should be informed about it (*p* = 0.028), (c) informing pregnant women is necessary/useful (*p* = 0.023), and (d) pregnant women showed interest in it (*p* = 0.034). Also, freelance midwives were more likely to believe that pregnant women should be informed about exercise (*p* = 0.006), and that they showed interest in it (*p* = 0.034). In addition, (a) freelance midwives (*p* = 0.050), and those who had experience in antenatal counselling (*p* = 0.037), as well as (b) obstetricians who were largely experienced in monitoring normal pregnancies (*p* = 0.001), were less likely to associate exercise during pregnancy with the occurrence of placental abruption. Conclusion: Alongside healthcare professionals’ educational level, their professional setting and professional experience emerge as key factors and need to be considered when designing innovative interventions to support exercise during pregnancy.

## 1. Introduction

During pregnancy, many women either do not engage in any physical activity program or significantly limit their physical activity, despite this period being described as an ideal time to provide education and motivation for pregnant women to start, improve, or increase their physical activity [1]. International guidelines for exercise during pregnancy recommend that healthcare professionals encourage pregnant women without medical contraindications and physically inactive women, as well as obese pregnant women, to gradually adopt a physical activity program to achieve optimal results, not only for their individual health but also for the health of their baby [2].

The international literature shows that healthcare professionals specializing in providing appropriate antenatal care to pregnant women, including midwives and obstetricians, play a crucial role in shaping their views and attitudes towards lifestyle issues such as physical exercise during this period [3,4]. They inform, advise, counsel, convey messages, and often encourage or discourage pregnant women to adopt certain health patterns [3,4,5]. Their role is therefore considered particularly important [6,7], as they are the ones who should provide accurate and reliable information to pregnant women about antenatal exercise (type, intensity, frequency, duration), aiming to improve their understanding of its beneficial effects and thus increase their participation in antenatal exercise programs. 

Most obstetricians assert that they take into serious consideration the significant benefits of regular exercise throughout pregnancy on the health of pregnant women and newborns, such as reducing the risk of gestational diabetes mellitus, excess weight gain, postpartum depression, and birthing a large-for-gestational-age infant [8,9]. Hayman et al. [9] found that the proportion of obstetricians who accepted the beneficial effects of exercise on maternal health was as high as 90%, while the proportion of those who accepted its benefits on neonatal health reached 70%. However, although obstetricians recognized the benefits of exercise during pregnancy, it was found that very few of them advised pregnant women about exercise on a regular basis during antenatal visits (24%) [8], while the same healthcare professionals reported at a high rate (56%) that pregnant women were usually the ones who asked and initiated the conversation about exercise [10]. According to Bauer et al. [11], these healthcare professionals provided little to no advice to pregnant women, and the time they spent on this topic during antenatal visits was very limited (about 5–10 min). In another survey, only 18% of obstetricians claimed to provide personalized advice to each pregnant woman [10]. All of these findings were consistent with and supported by statements from pregnant women as well, who have reported receiving zero to minimal guidance from their obstetricians on issues related to antenatal exercise [12]. Additionally, a large majority (83%) of obstetricians appeared to be unfamiliar with current international guidelines on exercise during pregnancy, while many of them may have had insufficient knowledge about its beneficial effects, such as on blood pressure response and pelvic floor strength [10].

Besides obstetricians, midwives, in their capacity as healthcare professionals, have a duty to inform, educate, and encourage pregnant women to lead a more active and healthier lifestyle during their pregnancy. The available research demonstrates that the majority of midwives have recognized and accepted the value of physical activity during pregnancy and its beneficial effects on both maternal and fetal health [13,14]. However, although midwives have claimed to be taking the benefits of exercise during pregnancy seriously, counselling pregnant women on antenatal exercise issues has not appeared to be a high priority compared to other issues they discussed with them antenatally [15]. Based on the existing literature, it is evident that midwives have often provided limited advice and guidance on exercise during pregnancy. Even when information has been provided, it has tended to be described as inadequate, unclear, or contradictory, often only being offered during the first antenatal appointment [5,14,15,16]. This cautious approach may lead to a lack of active encouragement for pregnant women to participate in exercise programs and, in some cases, even discourage them from starting or maintaining an antenatal exercise program. Furthermore, studies have highlighted that advice on antenatal exercise provided by midwives has frequently been limited and may not align with current international guidelines [5,14,16]. Additionally, such advice has often failed to accurately inform pregnant women about the recommended levels of intensity, frequency, and duration of exercise [5].

The reasons identified to explain the lack of counselling given by healthcare professionals regarding exercise were (a) insufficient education; (b) a lack of time on their part to provide counselling; (c) uncertainty about the effectiveness of providing counselling to women, i.e., uncertainty about whether they would eventually succeed in encouraging a pregnant woman to start or continue exercising, or embarrassment about how to initiate such a conversation (i.e., a lack of appropriate communication skills); and (d) their concerns about causing emotional distress to the pregnant woman [17], or concerns that potential miscommunication could damage their relationship with the pregnant woman, or affect her trust in them [17,18,19]. Admittedly, healthcare professionals have expressed a great willingness to actively participate in further education and training programs on antenatal exercise (e.g., through seminars or online courses) as a way to become more confident and more proactive in promoting pregnant women’s participation in exercise programs [10,17].

Taking into consideration that (a) physical activity and regular exercise during pregnancy have been identified as major factors for the improvement of the physical health of pregnant women, the fetus, and the newborn [20,21,22,23,24,25,26,27,28,29,30], and (b) the behavior of healthcare professionals attending to pregnant women can encourage or deter them from initiating or resuming an exercise program [31,32], it is particularly important to investigate these healthcare professionals’ perspectives and attitudes towards exercise during pregnancy, in order to examine the necessity of a series of measures and interventions from the state and scientific bodies that would focus on altering misconceptions and false beliefs around the issue and promoting exercise during this sensitive period of a woman’s life. Also, noting the lack of recent research at the international level on the critical factors that may undermine or act as a disincentive for the promotion of exercise by healthcare professionals, this study was designed and conducted with the aim of investigating the views and beliefs of Greek midwives and obstetricians regarding exercise during pregnancy. More specifically, this research aims to highlight the extent to which these healthcare professionals believe that (a) exercise during pregnancy is beneficial and necessary, (b) providing information to pregnant women is necessary/useful, (c) pregnant women are interested in exercise, and (d) exercise is associated with possible pregnancy complications. Also, this study aims to explore the socio-demographic and occupational factors that influence healthcare professionals’ views and beliefs on the issue.

## 2. Materials and Methods

### 2.1. Study Population

This is a cross-sectional study conducted during the period of January 2022–March 2023. The population of this study consisted of healthcare professionals (midwives and obstetricians), who, due to their training and professional rights, are the main providers of healthcare services throughout pregnancy. These healthcare professionals are responsible for monitoring and caring for pregnant women while providing information, advice and guidance to them, including information on exercise. This study was conducted on a sample of 237 Greek midwives and obstetricians, who worked in healthcare settings in Attica/Greece, namely (a) in two public general hospitals that are equipped with obstetric/midwifery clinics and receive the highest number of pregnant women seeking care in the prefecture; (b) in a large private hospital with general and obstetrics/midwifery clinics; (c) in primary public healthcare settings (1st Regional Health Authority of Attica); and (d) as freelancers. This research was conducted using convenience sampling and the sample was obtained from healthcare settings that were conveniently accessible. The selection of these specific healthcare settings allowed for the collection of a sample of healthcare professionals working at different levels of healthcare (primary, secondary/tertiary), and also in settings with different administrative characteristics (being either public-integrated in the national health system or private). 

### 2.2. Data Collection

**Figure 1:**
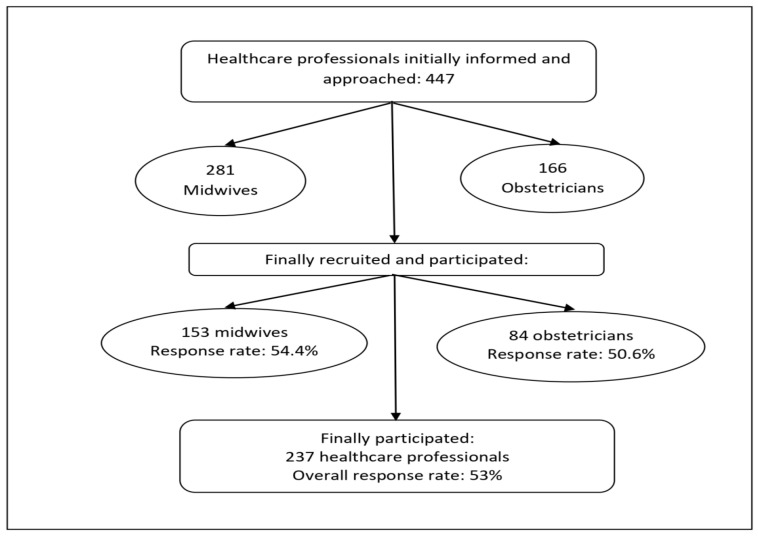
Participant information and recruitment.

All healthcare professionals who met the eligibility criteria (mentioned below) were approached and recruited in person by the primary researcher within their professional setting. The primary researcher was responsible for verbally informing the healthcare professionals, obtaining their written informed consent, distributing the questionnaire and receiving the completed form, and coding it and entering the information into a database. Initially, a total of 447 healthcare professionals (281 midwives and 166 obstetricians) were approached and verbally informed about the purpose and method of the survey by the researcher. Of those approached, 237 (153 midwives and 84 obstetricians) provided their written informed consent and eventually voluntarily participated in this study (53% response rate) (Figure 1). The eligibility criteria included the following: (a) having a midwifery and/or medical school degree (higher education), including professionals under training to become obstetrician specialists; (b) having a license to practice midwifery or medicine; and (c) having at least 1 year of professional experience in monitoring pregnant women. Midwifery and medical students were excluded. 

The healthcare professionals completed an anonymous, self-report questionnaire created for the purpose of this study. This questionnaire comprised the following sections: (a) socio-demographic and occupational information, (b) the healthcare professionals’ views and beliefs on the topic of exercise during pregnancy, (c) the practices they usually follow, and (d) their knowledge of international guidelines on the topic. The results from sections (c) and (d) are not presented in this article. Nine questions were used to assess the healthcare professionals’ demographics (i.e., gender, age, education level, specialty), and professional activity characteristics (i.e., experience, healthcare setting). The views and beliefs of the healthcare professionals were explored through asking them (a) to what extent they believed that exercise during pregnancy is beneficial in general, (b) to what extent they believed that informing pregnant women about the issue was necessary/useful, (c) to what extent they believed that the pregnant women they monitor were interested in exercise, and (d) to what extent they believed that exercise is associated with pregnancy complications in general. Each of these study variables was explored individually via a self-reported 5-point Likert scale, ranging from “not at all” to “to a very great extent”. In addition, as part of the investigation of their views and beliefs, the healthcare professionals were asked to indicate which they believed to be the main benefits of exercise and to note which complications they believed to be associated with exercise during pregnancy, out of 8 different options presented to them. 

Throughout the study, special attention was given to respecting the code of ethics of research and the principles of confidentiality and anonymity were considered. The questionnaire was anonymous. The participants and the hospitals in which they worked were coded by the researcher, and entered into a database (accessible only to the researcher). All necessary human research ethical approvals and permissions were granted from the scientific and ethics committees of (a) the 3 hospitals where the survey was conducted [Ref. Number (1st public hospital): 41/20-01-22, Ref. Number (2nd public hospital): 1480/28-01-22, and Ref. Number (private hospital): 9-12-2022], and (b) the 1st Regional Health Authority of Attica [Ref. Number: 21855/20-05-22]. All participants signed consent forms after being fully informed orally and in writing about the study’s purpose and methodology. 

### 2.3. Statistical Analyses

Quantitative variables were described as absolute frequencies (*n*) and relative frequencies (%). Continuous data were reported as mean values with standard deviations. Statistical significance was set at 0.05, while the minimal sample size required for each conducted statistical analysis was reached. Data analyses were performed using the Statistical Package for Social Sciences version 22.0. The demographic factors included gender, age, educational level, and specialty, while information on professional activity included professional experience (total), professional setting (public sector, private sector, primary care setting, freelance), professional position (director, attending doctor, resident, etc.), the extent to which they monitored normal pregnancies, and whether they participated or had participated in antenatal counseling programs (for midwives). 

The analyses of the relationships between the survey variables used the Pearson’s correlation coefficient (*r*), which determines the degree and type of correlation between two variables. Also, two analyses were performed using the Fisher’s Exact Test, as well as multiple analyses using the Mann–Whitney *U* statistical criterion for two independent samples and the Kruskal–Wallis statistical criterion for more than two independent samples. Finally, multiple analyses of variance were performed in order to investigate the relationship between the healthcare professionals’ beliefs/views towards exercise and the aforementioned socio-demographic and occupational characteristics. 

## 3. Results

### 3.1. Socio-Demographic and Occupational Characteristics of the Participants

Table 1 shows the socio-demographic and occupational characteristics of the 237 participants of the study. As can be noted, the participants were mainly female (75.10%, *n* = 178) compared to male (24.90%, *n* = 59), with the mean age of the participants being 40.65 years (*SD* = 11.11). Midwives comprised the majority of the sample (64.6%, *n* = 153) compared to the smaller proportion of obstetricians (35.4%, *N* = 84). A significant proportion had a postgraduate degree (38.4%, *n* = 91), and the mean number of years of professional experience among all of the healthcare professionals was 14.53 years (*SD* = 9.51). Regarding their professional setting, about half of them were working in a private hospital or as freelancers (52.8%, *n* = 125) and the rest were working in a public hospital or a public primary healthcare setting (47.3%, *n* = 112). Obstetricians who monitored normal (low-risk) pregnancies in 50–80% of their cases represented 49.9% (*n* = 41) of the sample, while those who monitored normal pregnancies in more than 80% of their cases constituted 37.4% (*n* = 31) of the sample. The vast majority of the midwives had experience in providing antenatal counselling to pregnant women (77.8%, *n* = 119), ranging mainly from one to ten years (81.4%, *n* = 92).

### 3.2. Healthcare Professionals’ Beliefs about the Benefits of Exercise during Pregnancy, and Associated Factors

**Figure 2:**
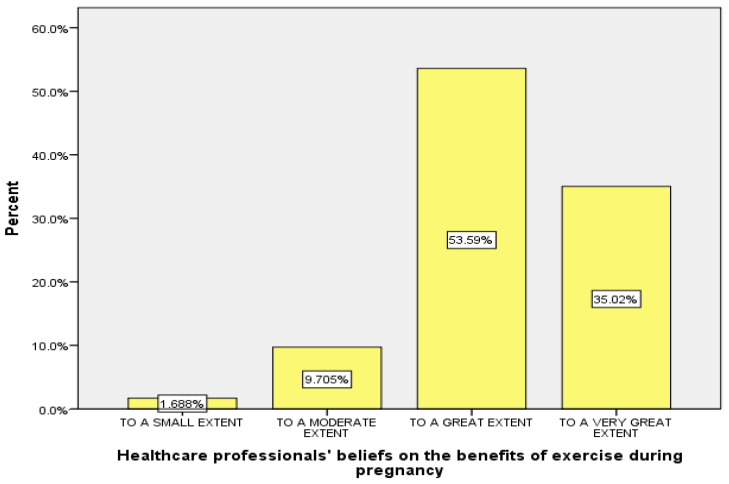
Healthcare professionals’ beliefs on the benefits of exercise during pregnancy.

**Figure 3:**
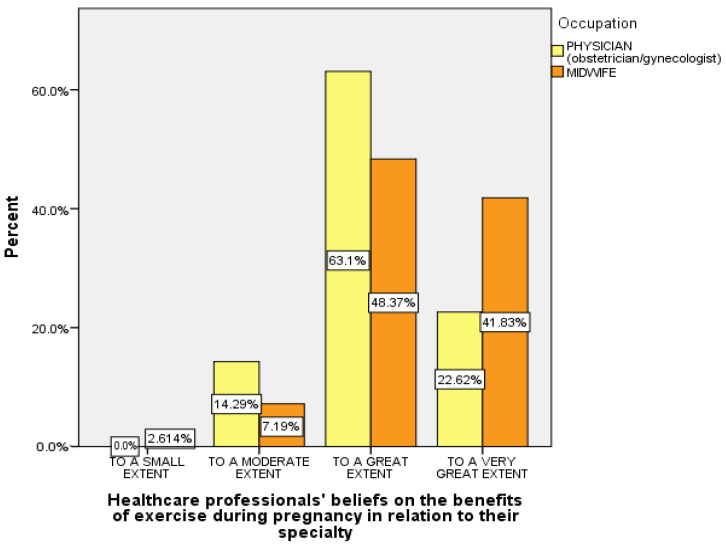
Healthcare professionals’ beliefs on the benefits of exercise during pregnancy in relation to their specialty.

As can be seen in Table 2, the vast majority of the healthcare professionals (88.6%) believed that exercise during pregnancy is generally beneficial, ranging from a great extent (53.6%) to a very great extent (35%) (also in Figure 2). According to the univariate analyses of variance, the *F* criterion (*df* = 1) showed a statistically significant relationship between the healthcare professionals’ beliefs about the benefits of exercise during pregnancy and (a) their specialty and (b) the obstetricians’ experience in monitoring normal pregnancies (Table 2). More specifically, it appears that exercise during pregnancy was considered beneficial by (a) midwives more than obstetricians (*p* = 0.032) (seen also in Figure 3) and (b) obstetricians who primarily monitored normal pregnancies (>80% of all pregnancies they monitored), more than those who monitored normal pregnancies to a lesser extent (50–80%) (*p* = 0.006). Furthermore, the Pearson’s correlation coefficient (*r*) between the healthcare professionals’ beliefs and their age was low and negative. Thus, it appears that the younger the age of the participants, the more they considered exercise during pregnancy to be beneficial (*r* = −0.156) (Table 2). Also, the Pearson’s correlation coefficient (*r*) (low, negative) indicated a possible relationship between the healthcare professionals’ beliefs on the necessity to inform women about exercise during pregnancy and their age. More specifically, the younger the participants’ age, the more they considered it necessary/useful to inform women about exercise during pregnancy (*r* = −0.133) (Table 2).

Table 3 presents data on the relationship between the healthcare professionals’ beliefs on the main benefits of exercise during pregnancy and their socio-demographic/occupational characteristics. As can be seen, characteristics such as gender, age, specialty, educational level, and total professional experience, the physicians’ experience in monitoring normal pregnancies, the midwives’ experience in providing antenatal counselling, and the professional setting (hospital/primary healthcare setting/freelance work) were statistically significantly associated with the participants’ beliefs on the main benefits of exercise during pregnancy.

More specifically, women (*p* = 0.036), midwives (*p* = 0.034), and obstetricians who usually monitored normal pregnancies (in 50–80% of their cases) (*p* = 0.045) were more likely to believe that exercise during pregnancy contributed to the prevention of excessive weight gain. Furthermore, the younger the participants’ age (*r* = −0.157) and the less professional experience they had (*r* = −0.171), the more they believed that exercise during pregnancy had this benefit for pregnant women. In addition, women (*p* = 0.046), midwives (*p* = 0.005), and those with postgraduate or doctoral degrees (*p* = 0.032) were more likely to believe that exercise during pregnancy was associated with a reduced risk of postpartum depression. Also, the younger the participants’ age, the more they believed that exercise during pregnancy had this benefit for women (*r* = −0.138).

Shorter labor stages as a benefit of exercise during pregnancy were mostly supported by women (*p* = 0.014), midwives (*p* = 0.001), and healthcare professionals with postgraduate or doctoral degrees (*p* = 0.028). Furthermore, the younger the participants’ age (*r* = −0.235) and the less professional experience they had (*r* = −0.190), the more likely they were to hold this belief. Additionally, women (*p* < 0.001), midwives (*p* < 0.001), and obstetricians who usually monitored normal (low-risk) pregnancies (50–80% of their cases) (*p* = 0.039) were more likely to believe in exercise causing an improved fetal descent into the pelvic tube (*p* = 0.039). Moreover, the younger the participants’ age, the more likely they were to hold this belief (*r* = −0.211).

The results also showed that those who believed that exercise improves musculoskeletal pains/discomfort in pregnant women were more likely to be women (*p* = 0.005), midwives (*p* < 0.001), those with postgraduate or doctoral studies (*p* = 0.045), and midwives with experience in antenatal counselling (*p* < 0.001). Also, the younger the participants’ age, the more likely they were to hold this belief (*r* = −0.158). Healthcare professionals with postgraduate or doctoral studies (*p* = 0.007) and freelance midwives (*p* = 0.012) were more likely to believe that exercise helped avoid or reduce hypertensive disorders in pregnant women. Furthermore, the younger the participants’ age (*r* = −0.137) and the greater their professional experience (*r* = 0.197), the more they believed this to be the case.

A reduced risk of gestational diabetes mellitus was most commonly reported as a benefit of exercise by healthcare professionals with postgraduate or doctoral degrees (*p* = 0.018) and midwives with experience in antenatal counselling (*p* = 0.039). Additionally, the greater the participants’ professional experience, the more they likely they were to hold this belief (*r* = 0.202). Moreover, healthcare professionals who had completed postgraduate or doctoral studies were more likely to support the improvement of the pregnant woman’s physical health as one of the benefits of exercise during pregnancy (*p* = 0.033). 

### 3.3. Healthcare Professionals’ Views about Whether Pregnant Women Are Interested in Exercise, and Associated Factors 

According to the analyses presented in Table 4, the *F* criterion shows a statistically significant relationship between the healthcare professionals’ view that the pregnant women they monitored were interested in exercise during pregnancy and several independent variables, such as (a) the healthcare professionals’ educational level, (b) the healthcare setting, (c) the obstetricians’ professional positions, and (d) the midwives’ experience in antenatal counselling. Specifically, healthcare professionals who were more likely to believe that pregnant women were interested in exercising included (a) freelancers or those who worked in private hospitals, compared to those in public hospitals or public primary healthcare settings (*p* < 0.001); (b) freelance obstetricians, compared to other obstetricians (*p* < 0.001); and (c) midwives with experience in antenatal counseling programs, compared to those without such experience (*p* = 0.001).

Table 5 presents the results of the Fisher’s Exact Test analyses of the relationship between the midwives’ views on whether pregnant women should be informed about exercise and their socio-demographic/occupational characteristics. As shown, (a) midwives who were freelance, and (b) those who had experience in antenatal counselling programs (current experience and/or past experience), were more likely to believe that all pregnant women should be informed about exercise during pregnancy, compared to those who worked in hospitals (public and private) and public primary healthcare settings (*p* = 0.028), and those with no experience in antenatal programs (*p* = 0.005), respectively.

### 3.4. Multivariate Analyses of Variance on Healthcare Professionals’ Beliefs and Views Regarding Exercise during Pregnancy

This section presents the results of four separate/independent models of multivariate analyses of variance designed to investigate the socio-demographic and occupational characteristics associated with healthcare professionals’ views and beliefs on exercise during pregnancy (Table 6). As shown, the *F* criterion (*df* = 1) revealed a statistically significant relationship between the healthcare professionals’ educational level and the extent to which they believed that (a) pregnant women should be informed about exercise (*p* = 0.010), (b) pregnant women are interested in exercise (*p* = 0.005), (c) exercise during pregnancy is beneficial in general (*p* = 0.003), and (d) it is necessary/useful to inform women about exercise during pregnancy (*p* = 0.019). More specifically, healthcare professionals with a postgraduate or doctoral education compared to those with only undergraduate degrees were more likely to believe that (a) pregnant women should be informed about exercise, (b) pregnant women show interest in it, (c) exercise during pregnancy is beneficial in general, and (d) it is necessary/useful to inform women about this topic. In addition, according to the *F* criterion (*df* = 1), the four different/independent multivariate analysis of variance models revealed a statistically significant relationship between the midwives’ views and beliefs on exercise during pregnancy and their professional setting. It appears that, compared to those who worked in hospitals (public and private) and public primary healthcare settings, freelance midwives were more likely to believe that (a) pregnant women should be informed about exercise (*p* = 0.006) and (b) pregnant women show an interest in exercise (*p* = 0.034).

### 3.5. Healthcare Professionals’ Beliefs on Whether Exercise during Pregnancy Is Associated with the Occurrence of Pregnancy Complications

Table 7 presents the results of analyses investigating the relationship between the healthcare professionals’ beliefs on whether exercise during pregnancy is associated with the occurrence of pregnancy complications and various socio-demographic/occupational characteristics. It appears that women were more likely than men to believe that exercise during pregnancy is generally associated with various pregnancy complications (*p* = 0.032). Furthermore, healthcare professionals working in primary healthcare settings were more likely to believe that exercise during pregnancy is associated with low-birth-weight infants (*p* = 0.012). Also, (a) midwives working in hospitals (public and private) and primary healthcare settings (public), (b) obstetricians who monitored normal pregnancies in 50–80% of their cases, and (c) midwives who did not have experience in antenatal counselling programs were more likely to believe that exercise during pregnancy is associated with the potential for placental abruption compared to freelance midwives (*p* = 0.050), obstetricians who monitored normal pregnancies to a greater extent (>80% of their cases) (*p* = 0.001), and midwives who did have experience in antenatal counselling (*p* = 0.037), respectively. 

## 4. Discussion

Our study aligns with previous research, indicating that a majority of healthcare professionals recognize the benefits of exercise during pregnancy for pregnant women and their fetuses [21,22,23,24,25,26,27,28,29,30]. However, our study expands upon these findings by conducting multivariate analyses, revealing that healthcare professionals’ educational levels significantly influence their beliefs and views regarding exercise during pregnancy. Specifically, midwives and obstetricians with postgraduate or doctoral degrees were more inclined to perceive exercise as generally beneficial and view providing information to pregnant women as necessary and valuable. This finding holds significant importance, considering the pivotal role healthcare professionals play in influencing women’s behaviors, particularly during pregnancy. Studies have demonstrated that pregnant women who receive physical activity and exercise counseling during antenatal visits exhibit higher levels of physical activity compared to those who do not receive such guidance [33,34,35]. Given that midwives, obstetricians, and general practitioners hold considerable influence over women’s decisions [31,32,36], while also serving as a trusted source of information [6,7], our study underscores the critical role of their educational level in shaping their views and beliefs.

Pregnancy represents a pivotal period for implementing interventions aimed at fostering healthier behaviors and altering existing patterns [37]. This phase offers a unique “window of opportunity” for intervention due to the sustained contact and provision of healthcare services over an extended period. Health education programs and interventions during pregnancy present numerous opportunities to promote healthy lifestyle patterns and enact preventive measures to mitigate maternal and infant morbidity [38,39]. Consequently, it is crucial to identify the factors associated with healthcare professionals’ views and beliefs regarding exercise during pregnancy.

In addition to the healthcare professionals’ educational levels, our study found that their professional settings significantly influenced their views and beliefs about exercise. It was therefore found that freelance midwives were more likely to believe that pregnant women should be informed about exercise, and that they show interest in it, compared to their counterparts in hospital (public and private) and public primary healthcare settings. This disparity may be attributed to the inherent differences in these work environments. Professionals in more rigid settings, such as hospitals or primary healthcare facilities, often face greater time constraints, necessitating adherence to strict schedules. Perhaps a professional setting that allows for more flexible working hours (e.g., longer antenatal visits); aims mainly at preventing pathology by recognizing its value; tailors the provision of antenatal counseling based on an assessment of the needs and circumstances of each woman/family, as recommended [40]; and prioritizes the provision of antenatal counseling on lifestyle patterns among pregnant women would facilitate the promotion of healthy patterns/behaviors (such as exercise).

At the same time, such promotion could be facilitated through the introduction of institutional measures, including the systematic training and empowerment of healthcare professionals [16], the enhancement of undergraduate programs in midwifery, obstetrics, and medicine [8,16,41], and the integration of antenatal physical activity and exercise into antenatal care services [16]. Additionally, providing individualized counseling [16], tailored to each woman’s personal needs and wishes, and prescribing exercise in specific cases [8], such as for obese and overweight women or physically inactive pregnant women, could further support these efforts. Moreover, the establishment or strengthening of community support facilities that adopt a multidisciplinary approach would ensure that counseling and guidance services meet the specific needs of each pregnant woman and those planning to conceive [42]. Funding interventions for vulnerable populations, such as migrants, who often adhere to cultural patterns, and aiming to change their attitudes towards exercise, is also crucial [43]. Implementing universal screening of every woman’s intention to exercise during early antenatal visits or incorporating this discussion into medical history-taking can provide valuable information and facilitate the initiation of these conversations [43]. These strategies collectively would promote healthier behaviors during pregnancy [15].

So far, the fear among healthcare professionals that exercise is associated with pregnancy complications has not been thoroughly investigated. This study suggests that both their professional setting and their professional experience influence healthcare professionals’ beliefs on this issue. Specifically, healthcare professionals working in more flexible settings, such as freelancers, and those with extensive experience in providing antenatal counseling and monitoring normal pregnancies are more likely to believe that exercise during pregnancy is only minimally associated with serious complications (e.g., placental abruption). Given that the fear of adverse outcomes often inhibits action, this finding is particularly important. It highlights the necessity of creating a framework that enhances the education, empowerment, and support of healthcare professionals, so that they can in turn create conditions of education and support for pregnant women and create incentives to encourage them to exercise [16]. 

Therefore, in addition to the previously reported barriers relating to a lack of education and time [40,44], the fear among professionals that exercise may cause pregnancy complications can be a significant impediment to promoting exercise during pregnancy. Since such fears often stem from a lack of education and knowledge—well-known barriers to providing counseling to pregnant women [15]—future research should investigate whether targeted and specific training and skills development could help reduce this fear and further motivate healthcare professionals towards providing antenatal counseling to all pregnant women. Addressing this issue is essential to reverse the current alarming trend of minimal or no advice on physical activity and exercise being provided to pregnant women, even in cases where additional motivation is necessary, such as with overweight or obese women [45] or those who were not physically active prior to their pregnancy [16,45].

To our knowledge, this is the first study in Greece to investigate the beliefs and views of healthcare professionals, specifically midwives and obstetricians, regarding exercise during pregnancy. Given the lack of recent studies that aim to identify the factors influencing healthcare professionals’ beliefs and views on exercise, and considering the significant benefits of exercise for pregnant women and their fetuses, such research is particularly important. These findings should be taken into account when designing and implementing innovative interventions and support programs to promote appropriate health-related practices and behaviors. Furthermore, this study holds great value as it was conducted in a country with a unique obstetric context, where obstetricians, rather than midwives, typically provide counseling and monitor the progress of pregnancies, even in cases of perfectly normal pregnancies. 

Nevertheless, this study has some limitations. The sample is not strictly representative of all Greek midwives and obstetricians. However, it includes healthcare professionals from two public hospitals that receive the highest number of pregnant women in Attica, Greece, public primary healthcare settings offering midwifery and obstetric services, and a large private hospital in the same region. Freelance healthcare professionals were approached through their collaboration with the private hospital. Given the large sample size and the inclusion of professionals from diverse settings, and considering that 50% of the Greek population resides in Attica, our results can be considered reliable and reflective of the beliefs and views of most midwives and obstetricians in this country. Another limitation may be that these findings are based exclusively on the self-reported beliefs and views of midwives and obstetricians. This raises concerns about the extent to which their responses were honest and free from social-desirability bias. Despite this, the use of self-reported data is a common and reliable method in many international surveys, provided that strategies to minimize response biases are carefully implemented in the study design and sample collection.

## 5. Conclusions

This study suggests that most healthcare professionals (midwives and obstetricians) in Greece recognize the benefits of exercise during pregnancy for both pregnant women and their fetuses, as well its necessity and positive effects. This study also indicates that for healthcare professionals, their educational level, professional setting, specialty, age, and experience in antenatal counselling and monitoring low-risk pregnancies are significantly associated with their beliefs and views on the benefits and necessity of exercise during pregnancy. Additionally, factors such as gender, professional setting, and professional experience appear to influence midwives’ and obstetricians’ beliefs on the likelihood of pregnancy complications being caused by exercise. Therefore, further research in this field is necessary, and all of the aforementioned factors should be considered when designing innovative interventions and strategies aimed at promoting healthy behaviors during pregnancy and improving the health status of pregnant women and their fetuses. 

## Figures and Tables

**Table 1:** Participants’ socio-demographic and occupational characteristics.

	*n*/*M*	%/*SD*
Gender		
Male	59	24.9
Female	178	75.1
Total	237	100.0
Age	40.65	11.11
Specialty		
Obstetrician	84	35.4
Midwife	153	64.6
Total	237	100.0
Educational level		
Undergraduate	127	53.6
Postgraduate	91	38.4
Doctorate	18	8.0
Total	236	100.0
Professional experience (years)	14.53	9.51
1–5 years	42	19.0
6–10 years	57	25.8
11–15 years	32	14.5
16–20 years	32	14.5
>20 years	58	26.2
Total	221	100.0
Professional setting		
Public hospital	72	30.4
Public primary care setting	40	16.9
Private hospital	31	13.1
Self-employed	94	39.7
Total	237	100.0
Obstetricians’ professional position		
Director	6	7.1
Curator	14	16.7
Obstetrics Specialist	17	20.2
Professor	1	1.2
Self-employed	46	54.8
Total	84	100.0
Obstetricians’ experience in monitoring normal (low-risk) pregnancies		
>80% of all monitored pregnancies	31	37.4
50–80% of all monitored pregnancies	41	49.4
30–50% of all monitored pregnancies	9	10.8
1–30% of all monitored pregnancies	2	2.4
Total	83	100.0
Midwives’ experience in providing antenatal counselling to pregnant women (now or in the past)		
No	34	22.2
Yes	119	77.8
Total	153	100.0
Midwives’ years of experience in providing antenatal counselling to pregnant women (years)	7.19	6.37
1–3 years	39	34.5
4–10 years	53	46.9
>10 years	21	18.6
Total	113	100.0

Note. *n*—frequencies; *M*—Mean; %—relative frequencies; *SD*—Standard Deviation.

**Table 2:** Investigation of healthcare professionals’ beliefs regarding the benefits of exercise during pregnancy and the necessity to inform women about this issue, in relation to their socio-demographic/occupational characteristics.

Healthcare Professionals Who Believed that Exercise during Pregnancy is Beneficial for Pregnant Women in General	*M*	*F*(1)	*p*	*η* ^2^
Specialty				
Obstetrician	4.08	4.665	0.032	0.020
Midwife	4.26			
Obstetricians’ experience in monitoring normal (low-risk) pregnancies				
>80%	4.35	8.051	0.006	0.103
50–80%	3.98			
	Pearson’s *r*			
Healthcare professionals’ age	−0.156 *			
**Healthcare professionals who believed that it is necessary/useful to inform women about exercise during pregnancy**				
	Pearson’s *r*			
Healthcare professionals’ age	−0.133 *			

Note: * = The correlation is significant at the 0.05 level (two-tailed test).

**Table 3:** Investigating the relationship between health professionals’ views/beliefs on the main benefits of exercise and their socio-demographic/occupational characteristics.

Healthcare Professionals’ Perceptions of the Main Benefits of Exercise in Pregnancy:				
(A) Prevention of excessive weight gain (Mann–Whitney *U*)				
Gender	*n*	Mean Rank	*U*	*p*
Male	59	105.78	4471.00	0.036
Female	178	123.38		
Specialty				
Obstetrician	84	108.63	5554.50	0.034
Midwife	153	124.70		
Obstetricians’ experience in monitoring normal (low-risk) pregnancies				
>80%	31	31.60	483.500	0.045
50–80%	41	40.21		
	Pearson’s *r*			
Age	−0.157 *			
Professional experience	−0.171 *			
(Β) Reduced risk of developing postpartum depression (Mann–Whitney *U*)				
Gender	*n*	Mean Rank	*U*	*p*
Male	59	105.68	4465.00	0.046
Female	178	123.42		
Specialty				
Obstetrician	84	104.41	5200.50	0.005
Midwife	153	127.01		
Educational level				
Undergraduate	127	110.89	5954.50	0.032
Postgraduate/doctorate	109	127.37		
	Pearson’s *r*			
Age	−0.138 *			
(C) Shorter duration of the labor stage (Mann–Whitney *U*)				
Gender	*n*	Mean Rank	*U*	*p*
Male	59	102.64	4286.00	0.014
Female	178	124.42		
Specialty				
Obstetrician	84	102.36	5028.00	0.001
Midwife	153	128.14		
Educational level				
Undergraduate	127	110.74	5936.00	0.028
Postgraduate/doctorate	109	127.54		
	Pearson’s *r*			
Age	−0.235 **			
Professional experience	−0.190 **			
(D) Improvement in the progression of fetal descent through the pelvic tube (Mann–Whitney *U*)				
Gender	*n*	Mean Rank	*U*	*p*
Male	59	93.21	3129.50	<0.001
Female	178	127.55		
Specialty				
Obstetrician	84	95.20	4425.50	<0.001
Midwife	153	132.07		
Obstetricians’ experience in monitoring normal (low-risk) pregnancies				
>80%	31	31.63	484.500	0.039
50–80%	41	40.18		
	Pearson’s *r*			
Age	−0.211 **			
(Ε) Improvement of musculoskeletal pains/discomfort in pregnant women (Mann–Whitney *U*)				
Gender	*n*	Mean Rank	*U*	*p*
Male	59	100.73	4173.00	0.005
Female	178	125.06		
Specialty				
Obstetrician	84	100.11	4839.00	<0.001
Midwife	153	129.37		
Educational level				
Undergraduate	127	111.54	6037.50	0.045
Postgraduate/doctorate	109	126.61		
Midwives’ experience in antenatal counseling programs (Kruskal–Wallis)	*n*	Mean Rank	*χ*^2^(3)	*p*
No	34	50.50	29.565	<0.001
Yes, at the moment	27	71.67		
Yes, in the past	37	89.66		
Yes, both at the moment and in the past	55	87.48		
	Pearson’s *r*			
Age	−0.158 *			
(F) Avoidance/reduced possibility of hypertensive disorders in pregnant women (hypertension, pre-eclampsia, etc.) (Mann–Whitney *U*)				
Educational level	*n*	Mean Rank	*U*	*p*
Undergraduate	127	109.02	5718.00	0.007
Postgraduate/doctorate	109	129.54		
Professional setting				
Hospital (public/private)/public primary healthcare setting	40	51.15	1226.000	0.012
Freelance work	81	65.86		
	Pearson’s *r*			
Age	−0.137 *			
Professional experience (in years) (continuous variable)	0.197 *			
(G) Reduced risk of developing gestational diabetes mellitus (Mann–Whitney *U*)				
Educational level	*n*	Mean Rank	*U*	*p*
Undergraduate	127	110.10	5854.50	0.018
Postgraduate/doctorate	109	128.29		
Midwives’ experience in antenatal counseling programs (Kruskal–Wallis)	*Ν*	Mean Rank	*χ*^2^(3)	*p*
No	34	67.50	8.381	0.039
Yes, at the moment	27	84.17		
Yes, in the past	37	81.49		
Yes, both at the moment and in the past	55	76.34		
	Pearson’s *r*			
Professional experience (in years) (continuous variable)	0.202 *			
(H) Improvement of the pregnant woman’s physical health (Mann–Whitney *U*)				
Educational level	*n*	Mean Rank	*U*	*p*
Undergraduate	127	111.83	6075.00	0.033
Postgraduate/doctorate	109	126.27		

Note: ** = the correlation is significant at the 0.01 level (two-tailed test); * = the correlation is significant at the 0.05 level (two-tailed test).

**Table 4:** Healthcare professionals’ views/beliefs regarding pregnant women’s interest in exercise during pregnancy in relation to their socio-demographic/occupational characteristics.

Healthcare Professionals Who Considered That Pregnant Women Were Interested in Exercise during Pregnancy				
Professional setting	*M*	*F*(1)	*p*	*η* ^2^
Public hospital	2.67 a	7.339	<0.001	0.087
Public primary healthcare setting	2.62 a			
Private hospital	3.13 b			
Freelance work	3.10 b			
Obstetricians’ professional position	*M*	*F*(2)	*p*	*η* ^2^
Director/Curator/Professor	2.52 a	9.428	<0.001	0.191
Obstetrics Specialist	2.65 ab			
Freelancer	3.20 b			
Midwives’ experience in antenatal counselling programs	*M*	*F*(1)	*p*	*η* ^2^
No	2.45	12.259	0.001	0.076
Yes	2.99			

Note. Means that do not share a common index (a, b) are significantly different from each other, according to Scheffé post-hoc test for *α* = 0.05.

**Table 5:** Midwives’ views on whether pregnant women should be informed about exercise, in relation to their socio-demographic/occupational characteristics.

Midwives Who Considered That Pregnant Women Should Be Informed about Exercise:	Yes, All Pregnant Women		Yes, Only Low-Risk Pregnant Women		Yes, Only Pregnant Women Who Were Interested in Exercise		Yes, Only Pregnant Women Who Exercised before Pregnancy		Fisher’s Exact Test	*p*
	C.	E.C.	C.	E.C	C.	E.C.	C.	E.C		
Professional setting										
Hospital (public, private)/primary healthcare setting (public)	30	34.0	8	5.0	2	0.7	0	0.3	7.298	0.028
Freelance work	73	69.0	7	10.0	0	1.3	1	0.7		
Midwives’ experience in antenatal counselling programs										
No	25	28.2	6	4.4	3	1.1	0	0.2	15.439	0.005
Yes, at the moment	18	22.4	7	3.5	1	0.9	1	0.2		
Yes, in the past	35	30.7	2	4.8	0	1.2	0	0.2		
Yes, both at the moment and in the past	49	45.7	5	7.2	1	1.8	0	0.4		

Note: C.—count, E.C.—expected count.

**Table 6:** Four separate/independent models of multivariate analyses of variance to explore the relationship between healthcare professionals’ beliefs and views on exercise during pregnancy and their socio-demographic and occupational characteristics.

**Model A: Healthcare professionals who believed that pregnant women should be informed about exercise during pregnancy**	*M*	*F*(1)	*p*	*η* ^2^
Educational level				
Undergraduate	4.09	6.692	0.010	0.028
Postgraduate/doctorate	4.33			
Midwives: Professional setting				
Hospital (public or private)/primary healthcare setting (public)	4.03	7.691	0.006	0.063
Freelance work	4.41			
**Model B: Healthcare professionals who considered pregnant women to be interested in exercise during pregnancy**	*M*	*F*(1)	*p*	*η* ^2^
Educational level				
Undergraduate	2.76	8.046	0.005	0.034
Postgraduate/doctorate	3.05			
Midwives: Professional setting				
Hospital (public or private)/primary healthcare setting (public)	2.75	4.581	0.034	0.037
Freelance work	3.07			
**Model C: Healthcare professionals who believed that exercise during pregnancy is beneficial in general**	*M*	*F*(1)	*p*	*η* ^2^
Educational level				
Undergraduate	4.11	8.751	0.003	0.03
Postgraduate/doctorate	4.37			
**Model D: Healthcare professionals who believed that it is necessary/useful to inform women about exercise during pregnancy**	*M*	*F*(1)	*p*	*η* ^2^
Educational level				
Undergraduate	3.93	5.535	0.019	0.023
Postgraduate/doctorate	4.13			

**Table 7:** Investigation of healthcare professionals’ beliefs on whether exercise during pregnancy is associated with the occurrence of pregnancy complications, in relation to their socio-demographic/occupational characteristics.

Healthcare Professionals Who Believed that Exercise during Pregnancy is Generally Associated with Pregnancy Complications				
Gender (Mann–Whitney *U*)	*n*	Mean Rank	*U*	*p*
Male	59	111.00	4779.00	0.032
Female	175	119.69		
**Healthcare professionals who believed that exercise during pregnancy is associated with low-birth-weight infants**				
Professional setting (Kruskal–Wallis)	*n*	Mean Rank	*x*^2^(3)	*p*
Public hospital	71	120.09	10.920	0.012
Primary healthcare setting (public)	39	125.50		
Private hospital	30	113.50		
Freelance work	94	113.30		
**Healthcare professionals who believed that exercise during pregnancy is associated with placental abruption**				
Professional setting (Mann–Whitney *U*)	*n*	Mean Rank	*U*	*p*
Hospital (public/private)/primary healthcare setting (public)	40	67.50	1320.000	0.050
Freelance work	80	57.00		
Obstetricians’ experience in monitoring normal (low-risk) pregnancies				
>80% of all monitored pregnancies	31	28.94	401.000	0.001
50–80% of all monitored pregnancies	40	41.48		
Midwives’ experience in antenatal counselling programs (Kruskal–Wallis)	*n*	Mean Rank	*x*^2^(3)	*p*
No	34	91.47	8.494	0.037
Yes, at the moment	27	65.89		
Yes, in the past	36	72.22		
Yes, both at the moment and in the past	55	75.25		

## Data Availability

Data are contained within the article.
